# Stochastic resonance in a proton pumping Complex I of mitochondria membranes

**DOI:** 10.1038/s41598-017-12746-0

**Published:** 2017-09-29

**Authors:** D. Kaur, I. Filonenko, L. Mourokh, C. Fendler, R. H. Blick

**Affiliations:** 1 Department of Physics, Queens College of the City University of New York, Flushing, NY 11367 USA; 2 Edgemont High School, Scarsdale, NY 10583 USA; 3 The Graduate Center of CUNY, New York, NY 10016 USA; 4 Center for Hybrid Nanostructures (CHyN) and Institutes of Nanostructure and Solid State Physics, University of Hamburg, Luruper Chaussee 149, 22761 Hamburg, Germany; 5 College of Engineering, Materials Science and Engineering, University of Wisconsin-Madison, 1509 University Avenue, Madison, WI 53706 USA

## Abstract

We make use of the physical mechanism of proton pumping in the so-called Complex I within mitochondria membranes. Our model is based on sequential charge transfer assisted by conformational changes which facilitate the indirect electron-proton coupling. The equations of motion for the proton operators are derived and solved numerically in combination with the phenomenological Langevin equation describing the periodic conformational changes. We show that with an appropriate set of parameters, protons can be transferred against an applied voltage. In addition, we demonstrate that only the joint action of the periodic energy modulation and thermal noise leads to efficient uphill proton transfer, being a manifestation of stochastic resonance.

## Introduction

One of the most important energy conversion mechanisms in nature are proton-pumping complexes of mitochondrial membranes. They enable to convert electronic energy into the more stable form of a proton gradient across a cellular membrane^1^. Though these complexes are well studied in biology, the actual physical mechanisms of energy conversion have remained elusive for many cases. This is especially true for Complex I^2^, where a tremendous distance of up to 15 nm separates electrons and protons, which suggests that a nanomechanical (conformational) mechanism is at play. The structure of bacterial Complex I was determined in refs^3–5^ and recently the studies of yeast^6^ and mammalian^7,8^ complexes exhibited similar structures. This particular enzyme consists of an L-shaped assembly of a hydrophobic arm embedded in the lipid membrane and a hydrophilic peripheral arm, which protrudes into the mitochondrial matrix. Electron transfer occurs in the hydrophilic domain via a set of FeS complexes, while the actual proton pumps are located within the membrane.

The mechanism of coupling between electron transfer and proton translocation is still enigmatic^8^. In the model proposed in refs^4,9^, it was suggested that the motion of the helix HL might be responsible for proton pumping. In the oxidized state, this helix moves to the right to open the upper half-channels for protons. In the reduced state, it moves to the left to open the lower half-channels. This model does not, however, explain what the energy transfer mechanism from the hydrophilic to the membrane domain is in order to assist proton transfer against the population gradient. Moreover, the crucial role of the helix in proton pumping was not confirmed in the mutation experiments^10^. Instead, it is proposed^11^ that stabilization of negatively charged quinone intermediates drives a conformational change. Two different conformations of the complex supporting this idea were found in recent experiments^6–8^.

Here, we propose a model for the indirect electron-proton coupling, assisted by conformational changes. It is well established that in all electron-driven proton pumps, the electron-proton Coulomb interaction plays a crucial role in transferring energy from electron to proton degrees of freedom. While in Complex I, electrons and protons are well separated. Consequently, direct Coulomb coupling seems to be unlikely. Rather, electron transfer events in the peripheral arm and at the hydrophilic-membrane domain interface^7^ facilitate periodic charge redistribution in the membrane domain. In particular, it was proposed^6^ that coordinated loops rearrangement could result in a shift of the ubiquinone binding site and a movement of a cluster of negative charges which, in turn, might trigger an electrostatic pulse toward the membrane arm. However, it is still unclear how this electrostatic pulse enables the uphill proton transfer. In the present work, we reveal this mechanism. We focus on the energy transfer between electron and proton subsystems, while neglecting the exact dynamics associated with the conformational changes. They are modeled by a positive charge *Q* = *e*, with a periodically changed distance to the proton sites. It should be noted that if the actual charge would be negative, it would not affect the results of our work.

**Figure 1:**
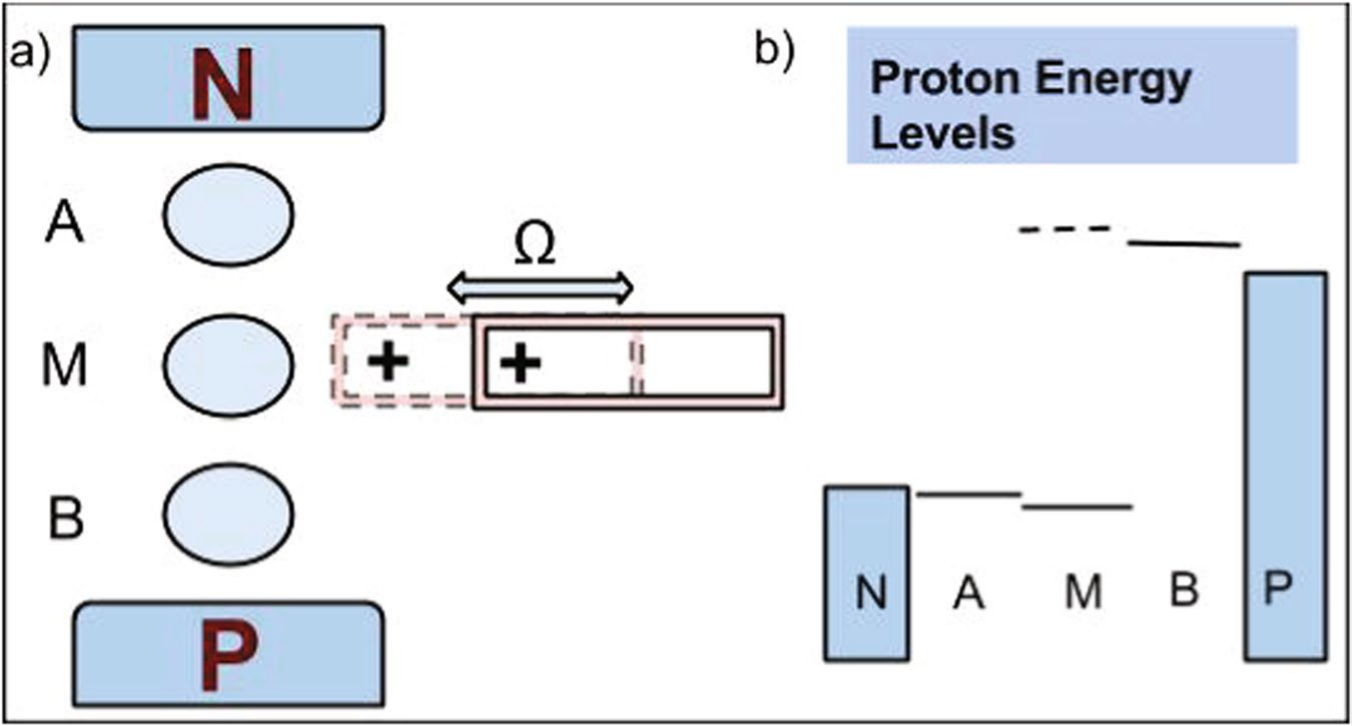
(**a**) Schematics of the model: three proton sites are placed between the source and drain reservoirs. The piston having a charge near the edge oscillates in the vicinity of the middle site. (**b**) Energy diagram: The energy of the site *A* is slightly below the chemical potential of the source, while the energy of the site *B* is slightly above the chemical potential of the drain. The solid line represents the energy of the site *M* when the piston is moved far away from the site and the dashed line shows the energy of this site when the piston returns back.

In the present paper, we discuss a model (see Fig. 1(a)), in which the electron transfer causes a periodic external force acting on the movable charge. This system consists of three proton sites (*A*, *B*, and *M*) located between the source and drain, and the charge located near the middle site *M*. The energies of the sites and the chemical potentials of the reservoirs are shown in Fig. 1(b). With the movable charge close to the site *M*, its energy is greater than that of site *B*, while when the charge is moved away, it is lower than that of the site *A*. The interaction of the system with the environment (represented as a set of independent oscillators) leads (i) to the reorganization of the environment due to the charge transfer events between the sites and (ii) to friction of the charge motion. Moreover, we show that the thermal noise caused by the environment is essential for an effective proton pumping.

## Theoretical Approach

The Hamiltonian of this system is given by

1 $$\begin{array}{rcl}H & = & {E}_{A}{a}_{A}^{+}{a}_{A}+{E}_{B}{a}_{B}^{+}{a}_{B}+{E}_{M}{a}_{M}^{+}{a}_{M}-{{\rm{\Delta }}}_{AM}{a}_{M}^{+}{a}_{M}-{{\rm{\Delta }}}_{AM}^{\ast }{a}_{A}^{+}{a}_{M}\\  &  & -{{\rm{\Delta }}}_{MB}{a}_{B}^{+}{a}_{M}-{{\rm{\Delta }}}_{MB}^{\ast }{a}_{M}^{+}{a}_{B}\\  &  & +\sum _{k}{E}_{Sk}{s}_{k}^{+}{s}_{k}+\sum _{k}{E}_{Dk}{d}_{k}^{+}{d}_{k}-\sum _{k}{T}_{Sk}{a}_{A}^{+}{s}_{k}\\  &  & -\sum _{k}{T}_{Dk}{a}_{B}^{+}{d}_{k}-\sum _{k}{T}_{Sk}^{\ast }{s}_{k}^{+}{a}_{A}-\sum _{k}{T}_{Dk}^{\ast }{d}_{k}^{+}{a}_{B}\\  &  & +\sum _{j}\frac{{p}_{j}^{2}}{2{m}_{j}}+\sum _{j}\frac{{m}_{j}{\omega }_{j}^{2}}{2}{({x}_{j}-{C}_{Aj}{a}_{A}^{+}{a}_{A}-{C}_{Bj}{a}_{B}^{+}{a}_{B}-{C}_{Mj}{a}_{M}^{+}{a}_{M})}^{2},\end{array}$$

where ${a}_{\sigma }^{+}/{a}_{6}$ are the proton creation/annihilation operators for the σ-site (σ = *A*, *B*, *M*), *E*_*σ*_ are the energies of these sites, Δ_σσ_’ are the transfer amplitudes between the sites, ${s}_{k}^{+}/{s}_{k}$ and ${d}_{k}^{+}/{d}_{k}$ are the creation/annihilation operators for protons with wave-vector *k* for the source and drain, respectively, *T*_*Sk*_ and *T*_*Dk*_ are the transfer magnitudes between the sites and the reservoirs, *p*_*j*_ and *x*_*j*_ are the momentum and coordinate of the *j*-th harmonic oscillator with mass *m*_*j*_ and frequency *ω*_*j*_, and *C*_*σj*_ are the coupling strengths of the proton-environment interaction.

The energy of the site *M* depends on the charge position *x* as

2 $${E}_{M}={E}_{M0}+\frac{{e}^{2}}{4\pi \varepsilon {\varepsilon }_{0}}\frac{1}{\sqrt{{({l}_{p}+x)}^{2}+{r}_{p}^{2}}},$$

where *l*_*p*_ is the horizontal distance between the charge at equilibrium and the site *M* and *r*_*p*_ is its vertical shift. We assume that the charge motion is in the overdamped regime. Thus, the charge position obeys the phenomenological Langevin equation,

3 $$\zeta \frac{dx}{dt}=-kx+\frac{{e}^{2}}{4\pi \varepsilon {\varepsilon }_{0}}\frac{{N}_{M}({l}_{p}+x)}{{({({l}_{p}+x)}^{2}+{r}_{p}^{2})}^{3/2}}+A(1+\,\cos \,{\rm{\Omega }}t)+\xi (t),$$

where *k* is the elastic force constant, *N*_*M*_ is the population of the *M*-site, *A* and Ω are the amplitude and frequency, respectively, of the periodic force associated with the electron transfer in the hydrophilic domain, *ζ* is the drag coefficient, and *ξ* is the fluctuation source (white noise) with zero mean value and the correlation function given by

4 $$\langle \xi (t)\xi (t\text{'})\rangle =2\zeta T\delta (t-t\text{'}).$$

Equations for the site populations can be derived using the equations of motion for the creation/annihilation operators of Eq. (1). It was shown previously^12–14^ that in the high-temperature limit the resulting rate equations can be written as

5 $$\begin{array}{c}\langle {\dot{N}}_{A}\rangle +{{\rm{\Gamma }}}_{S}\langle {N}_{A}\rangle ={{\rm{\Gamma }}}_{S}{F}_{S}({E}_{A})+{{\rm{\Phi }}}_{A},\\ \langle {\dot{N}}_{B}\rangle +{{\rm{\Gamma }}}_{D}\langle {N}_{B}\rangle ={{\rm{\Gamma }}}_{D}{F}_{D}({E}_{B})+{{\rm{\Phi }}}_{B},\\ \langle {\dot{N}}_{M}\rangle =-{{\rm{\Phi }}}_{A}-{{\rm{\Phi }}}_{B}.\end{array}$$

Here, angled brackets mean both quantum-mechanical and thermal averaging. Γ_*S*/*D*_ are the coupling constants to the reservoirs given by

6 $${{\rm{\Gamma }}}_{S/D}(\omega )=\sum _{k}{|{T}_{S/Dk}|}^{2}\delta (\omega -{E}_{S/Dk})$$

and assumed to be frequency-independent. *F*_*S*/*D*_ (*E*_*A/B*_) are Fermi functions for the protons at the reservoirs,

7 $$\begin{array}{rcl}{F}_{S}({E}_{A}) & = & \frac{1}{\exp \{({E}_{A}-{\mu }_{S})/T\}+1},\\ {F}_{D}({E}_{B}) & = & \frac{1}{\exp \{({E}_{B}-{\mu }_{D})/T\}+1},\end{array}$$

where *µ*_*S*/*D*_ are the chemical potentials of the source/drain. Kinetic coefficients Φ_*α*_ (*α* = *A*, *B*) have the form,

8 $${{\rm{\Phi }}}_{\alpha }={\kappa }_{\alpha }({E}_{\alpha }-{E}_{M}+{{\rm{\Lambda }}}_{\alpha })\langle {N}_{M}\rangle \langle 1-{N}_{\alpha }\rangle -{\kappa }_{\alpha }({E}_{M}-{E}_{\alpha }+{{\rm{\Lambda }}}_{\alpha })\langle {N}_{\alpha }\rangle \langle 1-{N}_{M}\rangle ,$$

where

9 $${\kappa }_{\alpha }(E)={|{{\rm{\Delta }}}_{\alpha M}|}^{2}\sqrt{\frac{\pi }{{{\rm{\Lambda }}}_{\alpha }T}}\exp \{-\frac{{E}^{2}}{4{{\rm{\Lambda }}}_{\alpha }T}\}$$

are the Marcus rates and

10 $${{\rm{\Lambda }}}_{\alpha }=\sum _{j}\frac{{m}_{j}{\omega }_{j}^{2}}{2}{({C}_{\alpha j}-{C}_{Mj})}^{2}$$

are the reorganization energies of the environment due to the proton transfer. The proton currents are given by

11 $$\begin{array}{rcl}{I}_{S} & = & \tfrac{d}{dt}\sum {s}_{k}^{+}{s}_{k}={{\rm{\Gamma }}}_{S}({N}_{A}-{F}_{S}({E}_{A})),\\ {I}_{D} & = & \tfrac{d}{dt}\sum {d}_{k}^{+}{d}_{k}={{\rm{\Gamma }}}_{D}({N}_{B}-{F}_{D}({E}_{B})).\end{array}$$

In the steady state regime, *I*_*S*_ = −*I*_*D*_. The proton pumping occurs when the drain current is positive. So, protons are transferred from the reservoir with lower chemical potential to the reservoir with higher chemical potential.

## Results and Discussion

**Figure 2:**
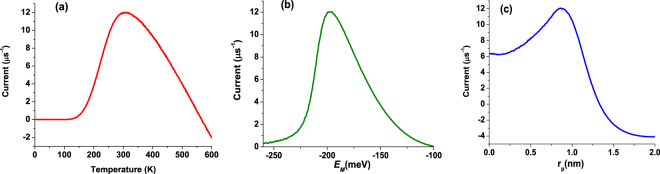
(**a**) The temperature dependence of the proton current; (**b**) dependence of the proton current on the unperturbed energy of the site *M*; (**c**) dependence of the proton current on the vertical shift of the piston with respect to the site *M*.

Eqs (4, 5) are coupled, as the electrostatic force acting on the piston depends on the population of the center site, while the energy of this site involved in Eqs (8, 9) depends on the piston’s position, as shown in Eq. (2). We solve these equations numerically, substituting the obtained values for the site populations in Eq. (11), and performing the time averaging and averaging over possible realizations of the white noise *ξ*. The results are shown in Fig. 2(a–c) for the following set of parameters: Ω = 10^9^ s^−1^, *A* = 41.4 nN, Λ_*A*_ = Λ_*B*_ = 50 meV, Δ_*AM*_ = Δ_*BM*_ = 25 meV, Γ_*S*_ = Γ_*D*_ = 10 meV, *l*_*p*_ = 0.5 nm, *k* = 8.9 N m^−1^, and *ζ* = 4.14 nN m^−1^ s, which corresponds to the diffusion coefficient *D* = *T*/*ζ* = 10^−12^ m^2^ s^−1^. The voltage applied across the membrane is 160 mV, so that the chemical potential of the source reservoir is assumed to be *μ*_*s*_ = −80 meV and the chemical potential of the drain reservoir is *μ*_*d*_ = 80 meV. The energy of the proton sites are *E*_*A*_ = −150 meV and *E*_*B*_ = 250 meV. These energies were chosen to prevent the back current at moderate temperatures, i.e., to ensure that the site *A* is always populated from the source reservoir and depopulated by the site *M*, and the proton is not transferred back to the reservoir. Correspondingly, the site *B* is always depopulated by the drain reservoir, see the energy levels in Fig. 1(b).

The temperature dependence of the current is shown in Fig. 2(a) for a vertical separation of *r*_*p*_ = 0.8 nm between the charge and the site *M* and the bare energy of the site *M* being *E*_*M*0_ = −200 meV. It is evident from this figure that for the chosen set of parameters, the most effective operation of the proton pump occurs at physiological temperatures. At high temperatures the current becomes negative because the broadening of the Fermi functions of the reservoirs enables the back current which becomes dominant. The bare energy of −200 meV of the site *M* is optimal, as can be seen from Fig. 2(b). The dependence of the current on the vertical shift is presented in Fig. 2(c). From the figure, we can assess that if the charge and the site *M* are well separated, the electrostatic energy is not enough to raise the energy of site *M* above that of site *B*. Instead, the energies of the sites become close in value and this enhances the back current.

**Figure 3:**
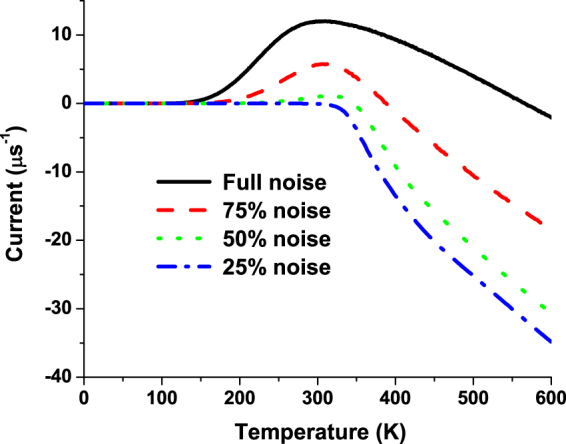
Temperature dependence of the pumped current for different noise levels and fixed magnitudes of the periodic force.

**Figure 4:**
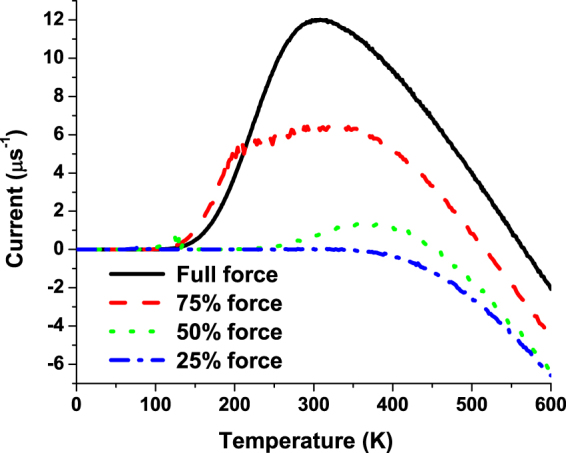
Temperature dependence of the pumped current for different magnitudes of the periodic force and fixed noise level.

It is evident from our analysis that the proton pumping in mitochondria membranes can be achieved by the three-site system when the energy of the center site is modulated by moving the charge which represents the periodic conformational changes. This effect is similar to the electron pumping achieved in semiconductor nanostructures: it was initially predicted by Thouless^15^ and later on experimentally verified^16^. The energy scales in these systems are almost 100 times smaller than that of the mitochondria, so the effects observed there at 4 K can take place at the physiological-temperature environment of mitochondria membranes. In refs^17,18^, another type of the charge pumping caused by a random force was predicted. This phenomenology is explored in various Brownian ratchets^19^. In our system, we have both periodically changed force and the white noise, so it is important to know what the origin of the proton pumping is. In Fig. 3, we show the temperature dependence of the pumped current for different levels of the white noise with the fixed magnitude of the periodic force. It is evident from this figure that the pumping disappears at low noise levels. Similar scenario can be seen in Fig. 4 where the level of the noise was fixed but the magnitude of the periodic modulation decreases. Correspondingly, we conclude that only the joint action of the periodic modulation and the noise can lead to the proton pumping in our model. This is the manifestation of stochastic resonance^20^ when the noise having a broad spectrum enhances the effect of the periodic driving. In our analysis, we have used the white noise, Eq. (4), with all the frequencies involved. The magnitude of this noise is quite large at the elevated physiological temperatures and even the moderate periodic driving force can be significantly amplified by means of the stochastic resonance.

## Summary

In summary, we have examined a possible mechanism of the proton pumping in Complex I of the mitochondria membranes relying on a moving-charge-mediated energy transfer process. Three proton sites are placed between the source and drain reservoirs with chemical potential of the source (negative side of the membrane) being smaller than the chemical potential of the drain (positive side). The moving charge representing the electron-driven conformational changes of the actual complex modulates the energy of the middle proton site. When the charge is far away, the energy of this site becomes smaller than the energy of the site near the source reservoir, so the center site is populated. When the charge returns back, the energy of the center site becomes larger than that of the site near the drain reservoir, and the proton is transferred there and eventually to the drain. Correspondingly, the proton pumping is achieved. We have shown that for a set of parameters similar to the real system, the operation of our model is most effective at physiological temperatures. Examining the physical origin of the obtained phenomenology, we have demonstrated that stochastic resonance, i.e. the joint action of the periodic modulation and the noise can lead to effective proton pumping.
